# Prevention of Dental Caries and Pyorrhea Alveolaris

**Published:** 1915-06

**Authors:** Stanley C. Lucas, Clarence M. Mote


### PREVENTION OF DENTAL CARIES AND PYORRHEA ALVEOLARIS.

READ BEFORE THE STUDENTS' DENTAL SOCIETY OF THE UNIVERSITY OF MICHIGAN, MAY 3, 1915.

BY STANLEY C. LUCAS AND CLARENCE M. MOTE.

It is an undisputed fact that the decay of teeth is due to the action of acid-forming bacteria. These bacteria must be confined to the teeth by means of plaques and must have carbohydrates upon which to work. This has been proven by many research workers, the first being Miller. To remove all bacteria has never been shown possible, but it is possible to remove the food, and the plaques which confine the bacteria to the teeth. The plaque is of fundamental importance. Either to prevent its formation or to remove it at least every twenty-four hours, is to prevent caries. It is not known why the plaque forms, nor how to prevent its formation; but we know how to successfully remove it. In some few cases its formation may be naturally inhibited, and if present research workers are successful, it may be possible sometime in the future to depend upon metabolism or upon some drug for immunity. Until such a time comes, we must depend upon our present accepted method of oral hygiene and prophylaxis, which most laymen, and many dentists, do not fully understand, nor appreciate.

The work of preventing caries and saving teeth, and avoiding many other troubles, does not fall wholly upon the dentist. The patient must do much faithful work, otherwise the honest efforts of the dentist amount to little or nothing. It is the duty of the dentist to intelligently instruct his patients regarding the care of the mouth and teeth. and to do such part of the care, as is not possible, or is not done by the patient. The remainder falls to the patient. It is essential that the patient should give proper care to the mouth and teeth *daily*. The quality and quantity of such care varies with each individual, but in order to be effective, must be sufficient to remove the plaques at least every twenty-four hours. Dr. O. E. Inglis said in a recent lecture, "The removal of the plaque is always indicated and it is the basis of prophylaxis. Can the plaque be removed and kept away? Probably not. Why, then, does prophylaxis, every one, two, or three months, together with proper daily care by the patient prevent caries and pyorrhea?

"Certified milk commissions admit that 10,000 bacteria to the cubic centimeter are practically harmless, while 500,000 become a source of great danger. This admits that numbers constitute a great factor, though, of course, the 10,000 may not be typhoid or such pathogenic bacteria. If, now, we consider the plaque as removed frequently, and, of course, frequently formed again, we must consider that the benefits of occasional prophylaxis arise from the fact almost universal in plant life, that frequent disturbances of the course of nutrition inhibits growth.

"Let us take a tree for example. Frequently transplanting into new soil, or in the case of grown trees, even one removal may cause the tree to die. The tree requires time to get set in the new location, to adjust its nutritive mechanism.

"We may look upon the plaque in like manner. The plaque requires at least some time, just how much can not be said, to become fixed, and the dominant organisms to produce characteristic detrimental effects on tooth or gum."

*Pyorrhea Alveolaris* is one of the most prevalent and worst diseases afflicting the human race. It is so wide spread, that some eminent authorities have claimed that a majority of all people over fifty years of age possess symptoms of pyorrhea to a greater or less degree. It is essentially a chronic disease, and runs a course extending over years, so that most people do not deem it injurious. The only apparent bad results as viewed by most people, is the loss of teeth, and this isn't regarded as of much importance. It is not a new disease, for it can be traced as far back as dental history goes. There seems to be no question but that pyorrhea is closely associated with some of our bad general diseases. Cummins says that "examination in many mild cases of pyorrhea alveolaris, not showing any special symptoms of intoxication, has demonstrated continued mild intoxication, manifested by depression, dyspeptic symptoms, and indigestion, and in the course of time the indigestion results in anemia and diminished resistance to bacterial invasion." He also says that "such cases may develop duodenal ulcers, gastro-enteritis, catarrhal conditions of the gall-duct, inflammation of the appendix, chronic diarrhea, and intestinal stasis."

Dr. William Osler believes that more physical degeneracy can be traced to neglect of the teeth than to the abuse of alcohol. Besides these disases, there are many others of the nervous, circulatory, and respiratory systems which have been shown to be largely due to oral sepsis. It can clearly be seen that the number of teeth lost because of pyorrhea is not nearly as important as the effect upon the general health. At the present time, much is said and written about pyorrhea, its cause, prevention, and treatment, both by dentists and physicians. There are a number of theories and views which seem to go to every extreme. There are some men who have gone so far as to say that the use of emetin hydrochloride alone, or an autogenous vaccine, will quickly cure all cases, and that in some cases, these remedies can be used without the assistance of the dentist or physician.

In order to determine whether or not pyorrhea is a curable disease, and what shall be the treatment, let us look further into the cause. According to the ablest and sanest diagnosticians, there are two classes of causes, the predisposing and exciting. The former includes such things as tartar formation, overhanging fillings, bad fitting crowns, and food between teeth, all of which irritate the tissues about the teeth. Among predisposing causes, are some of the constitutional diseases, as typhus, syphilis, rheumatism, tuberculosis, faulty metabolism, gout, and diabetes mellitis. Some of these diseases so weaken the resistance that the bacteria, which are the exciting cause, can gain a foothold. Age also seems to be a predisposing factor as it becomes more common with advancing age, above twenty-five or thirty years.

It is generally believed that a number of micro-organisms such as the endamoeba buccalis, pneumococcus, streptococcus, and certain pus-forming bacteria are the exciting cause of pyorrhea. Barrett and Smith, and some others, attribute the whole destruction of the peridental membrane and other tissues, to a single micro-organism, the endamoeba. If we believe that the predisposing causes named are important factors in causing this disease, we must also believe that removing these causes will prevent the disease, and that in trying to effect a cure after infection by the exciting causes, the first step must be to remove the predisposing causes, which can be done in most cases and unless far advanced, the teeth can be saved in a large percentage of cases. After having removed the predisposing causes by prophylaxis, oral hygiene, polishing fillings, general treatment, etc., which is the first essential, it is necessary in cases of advanced progress, to treat the exciting causes. The conservative treatment consists of using a mild antiseptic to destroy the bacteria and stimulate the formation of healthy granulations. In some bad cases more than a mild remedy may be necessary to cause a regeneration of tissues, and whenever the cause is systemic, it must be treated accordingly. Here, such remedies as emetin hydrochloride, autogenous vaccines, or the electrolytic treatment may prove helpful. Notwithstanding the claims that have been made regarding the wonderful curative properties of emetin, by prominent dentists and physicians, it seems only reasonable that it should be necessary to use the most rigid hygienic and prophylactic measures first, and along with the drug, so as to produce good and lasting results. Prevention of pyorrhea, like prevention of caries, is possible in nearly all cases, if the mouth is kept scrupulously clean, and all the teeth kept in the best possible condition. Regular visits to a good dentist are necessary. Here, if anywhere, ''an ounce of prevention is worth a pound of cure.'' A cure is also possible, unless the disease is far advanced. To check the disease, regardless of what the cause may be, the most effective way known is to thoroughly clean the mouth and teeth by mechanical and medicinal means, removing all local irritation caused by filings, crowns, roots, etc., treat systemic conditions when necessary, and cleanse the pus pockets with mild antiseptics so as to destroy the infection and stimulate healthy granulations.

